# Advances in research and adaptive expressions of entitlement: a mini review

**DOI:** 10.3389/fpsyg.2025.1689011

**Published:** 2025-10-30

**Authors:** Sivan George-Levi

**Affiliations:** Department of Psychology, Achva Academic College, Arugot, Israel

**Keywords:** entitlement, individual differences, narcissism, personality, social context

## Abstract

Entitlement, often defined as the belief in deserving special treatment or outcomes, has traditionally been seen as a pathological trait closely tied to narcissism and interpersonal dysfunction. However, accumulating evidence shows that entitlement is multidimensional and context-sensitive, with the potential to operate in both adaptive and maladaptive ways. This mini-review synthesizes advances across personality and social psychology, highlighting four developments: (a) recognition of distinct forms of entitlement, ranging from exploitative and inflated to active, assertive, and emotionally balanced; (b) growth of domain-specific research in relational, workplace, academic, and emotional contexts; (c) evidence that entitlement is activated by situational cues such as fairness, injustice, and life stress; and (d) identification of moderators that buffer risks and channel entitlement into constructive expressions. The findings indicate that the definition of entitlement has expanded beyond its original formulation, revealing a construct more complex than previously assumed, with empirical evidence showing varied and sometimes contradictory outcomes. Future research should prioritize multi-faceted and domain-targeted measures, employ longitudinal and experimental designs to clarify mechanisms, and apply integrative models that distinguish entitlement’s bright sides from its dark sides. These insights also hold practical relevance for education, organizations, relationships and therapeutic practice, where differentiating adaptive from maladaptive entitlement can guide more effective interventions.

## Introduction

We live in an age that increasingly emphasizes individual rights and personal needs over duty toward others (Passini and Renger, 2025). This shift is reflected in the concept of “entitlement,” often termed sense of entitlement or psychological entitlement, traditionally regarded as a maladaptive trait linked to narcissism, interpersonal conflict, and diminished well-being (Campbell et al., 2004; Freis and Hansen-Brown, 2021). Yet is entitlement necessarily harmful, or can certain entitlement beliefs and expectations also serve adaptive functions?

Recent research suggests that entitlement is a multifaceted construct (Golann and Darling-Aduana, 2020; Passini and Renger, 2025). Although it can promote conflict and frustration when expectations remain unmet, evidence also links entitlement to proactive workplace behavior, creativity, well-being, and the legitimate assertion of rights in sociocultural contexts (Sun et al., 2022; Zitek and Vincent, 2015; Lilly et al., 2025; Żemojtel-Piotrowska et al., 2017).

These findings mark a significant advance in reconceptualizing entitlement. Nonetheless, key questions remain regarding the mechanisms that exacerbate harm or facilitate adaptive expressions. Addressing these gaps requires a multidimensional, context-sensitive perspective that clarifies under what conditions entitlement functions as beneficial or detrimental (Renger and Passini, 2024; Yang et al., 2024). Unlike prior studies that mainly cataloged harmful correlates or focused on single domains, the present mini-review highlights the varied consequences of entitlement across contexts and integrates personality- and social-based perspectives.

In this mini-review, theoretical and empirical developments are synthesized, drawing on selected studies, including foundational work prior to 2000 that largely framed entitlement as a pathological trait, research in the early 2000s that established it as a distinct personality construct or as social expectation of fairness, and more recent contributions that emphasize multifaceted nature and contextual responsiveness.

These strands converge to illustrate four key advances: (a) recognition of multidimensionality; (b) emergence of domain-specific research across relational, workplace, academic, and emotional contexts; (c) evidence of contextual sensitivity, whereby situational conditions activate entitlement; and (d) identification of moderators that determine whether entitlement operates in adaptive or maladaptive ways. The objective of this review is to synthesize theoretical and empirical advances, clarify when entitlement is adaptive versus maladaptive, and provide best practice recommendations for future research.

The literature reviewed in this article was identified through searches in major scholarly databases (e.g., PsycINFO, PubMed, Web of Science) using keywords related to entitlement. In addition, AI-assisted tools (e.g., *Elicit*) were employed to support the identification of potentially relevant articles.

## Theoretical foundations and evolution of the concept

### Personality psychology perspective

Early research defined entitlement as a pervasive and stable belief that one deserves special treatment, rewards, or exemptions regardless of actual effort (Campbell et al., 2004). It was linked to narcissism, exploitative behavior, inflated self-esteem, a sense of superiority, and unrealistic expectations (Bishop and Lane, 2002; Twenge, 2006). Among narcissistic traits, entitlement and exploitativeness are the strongest predictors of aggression (Reidy et al., 2008).

While entitlement has often been described as a facet of narcissism, Campbell et al. (2004) argued that it should be regarded as a stand-alone personality trait, developing the Psychological Entitlement Scale (PES) to capture generalized beliefs of deservingness. The PES has been consistently linked to inflated expectations, interpersonal dysfunction, and difficulty with forgiveness (Exline et al., 2004; Exline and Zell, 2009; Hart et al., 2020).

Although entitlement and narcissism are correlated, evidence increasingly demonstrates their conceptual distinction (Campbell et al., 2004). Whereas narcissism reflects inflated self-views and the need for admiration (“I am great”), entitlement centers on expectations of preferential treatment (“I deserve more”). Unlike narcissism, which is centered on the self, entitlement reflects a paradoxical mix of dependence and independence oriented toward others (Rose and Anastasio, 2014).

Importantly, the PES is psychometrically distinct and predicts unique outcomes such as selfishness in resource dilemmas, aggression after criticism, and exploitative tendencies, above and beyond narcissism (Campbell et al., 2004; Rose and Anastasio, 2014). It shows strong retest reliability and correlates with grandiosity-related antagonism (especially immodesty), while also linking positively with non-exploitative entitlement and modestly with self-esteem (Ackerman and Donnellan, 2013). Entitlement has also been conceptualized as a cognitive-personality vulnerability that predisposes individuals to cycles of unmet expectations and distress not fully explained by narcissism (Grubbs and Exline, 2016).

Taking this personality-based perspective, entitlement has been associated with a wide range of negative outcomes, including unethical decision-making (Chen et al., 2023), rule-breaking (Zitek and Jordan, 2016), perceived inequity (Queiri and Alhejji, 2025), envy (Irshad et al., 2024), and chronic relationship conflict (Moeller et al., 2009). Entitled individuals are also more likely to experience anger and perceive injustice following random misfortunes (Zitek and Jordan, 2021).

A key development in the field is the recognition that entitlement cannot be reduced to a subdimension of narcissism but represents a distinct personality construct with unique correlates and contributions. While advances in personality research have been significant, this tradition largely treats entitlement as a stable trait rooted in problematic relational patterns (Grubbs and Exline, 2016), overlooking contextual influences and adaptive aspects, such as the legitimacy of asserting rights or the difficulties faced by those who lack sufficient entitlement. These dimensions are more clearly addressed within the social psychology perspective.

### Social psychology perspective

The social psychology perspective conceptualizes entitlement not as a fixed trait, but as a dynamic expectation of fairness and justice shaped by social norms and contextual factors (Feather, 2003; Lerner, 1987). As such, it reflects justified demands within social relationships and can be expressed either positively or negatively depending on the surrounding context (Feather, 2003; Tomlinson, 2013).

Extending this idea, Lewis and Smithson (2001) argued that entitlement was socially constructed and context-dependent, formed through the influence of gender norms, welfare policies, and workplace cultures. Entitlement was thus a response to structural conditions, such as access to work-life balance and career mobility, rather than an inherent personality trait.

Chatrakul Na Ayudhya and Smithson (2016) further challenged the stereotype of an “entitled” younger generation, suggesting that such expectations may be better understood as reflections of generational experiences and prevailing societal discourses. However, claims of generational differences in entitlement are not empirically supported. Recent large-scale studies and meta-analyses have refuted the notion of an ‘entitled generation,’ showing no systematic generational increases in narcissism or entitlement (Ravid et al., 2025; Weidmann et al., 2023).

Recent research highlights that entitlement does not always refer to excessive or self-serving demands. Passini and Renger (2025) distinguished between *over-entitlement*, marked by exaggerated claims, and *equal entitlement*, grounded in self-respect and the assertion of equal rights. Within this framework, the demands of disadvantaged groups for fair treatment may be understood as adaptive expressions of entitlement (Lilly et al., 2025). Although close to notions of fairness, this perspective still fits within the psychological definition of entitlement as a subjective belief in what one’s deserve.

Accordingly, the social psychology perspective emphasizes contextual triggers and adaptive functions, yet tends to overlook the stable and pervasive aspects highlighted in personality research. The following section therefore presents advances in research and integrative models that conceptualize entitlement as a multi-faceted construct bridging these approaches.

## Emerging perspectives on entitlement: theoretical integration and evidence of maladaptive and adaptive functions

Entitlement has been defined differently across traditions: in social psychology as the expectation of fair rewards for one’s efforts (Feather, 2003), and in personality psychology as a stable, sometimes excessive demand not grounded in actual contribution (Campbell et al., 2004). Rather than standing in opposition as in earlier work, personality and social perspectives are better understood as complementary, capturing both stable individual differences and context-dependent expressions of entitlement (Żemojtel-Piotrowska et al., 2017). From this view, entitlement encompasses both unrealistic claims of deservingness and legitimate expectations of positive outcomes.

It is important to note that while entitlement appears in the DSM-5 as a diagnostic criterion of narcissistic personality disorder (American Psychiatric Association, 2013), this review does not adopt a psychopathological lens. In personality and social psychology, both narcissism and entitlement are studied dimensionally as individual differences across the population, allowing exploration of adaptive as well as maladaptive forms (Pryor et al., 2008).

Research on entitlement has therefore undergone significant developments and shifts. First, integrating personality and social psychological perspectives, researchers increasingly view entitlement as a multidimensional (Żemojtel-Piotrowska et al., 2017). A key advancement has been the reconceptualization of entitlement into distinct dimensions, allowing for the identification of potentially healthy expressions of entitlement (Hart et al., 2020; Lessard et al., 2011; Tolmacz and Mikulincer, 2011).

Another major shift has been the recognition that entitlement is often domain-specific. It may manifest differently across life domains such as academic settings (e.g., Kinne et al., 2022), the workplace (e.g., Yang et al., 2024) interpersonal relationships (e.g., Tolmacz et al., 2021), and emotional functioning (e.g., Laslo-Roth and George-Levi, 2024). Third, research highlights the role of context in shaping how entitlement is expressed and perceived, emphasizing that situational factors may activate entitlement beliefs (Lilly et al., 2025). Finally, a growing body of work has identified moderating variables that determine whether entitlement functions adaptively or maladaptively (e.g., Klimchak et al., 2016; Langerud and Jordan, 2020; Schwarz et al., 2023; Yang et al., 2024).

### Multidimensional perspective on entitlement: theoretical approaches and measurement

Several studies have divided sense of entitlement into distinct dimensions. Table 1 provides an overview of selected instruments developed to assess entitlement. The table highlights their theoretical origins, domains of application, and dimensional structure. As can be seen in Table 1 and Lessard et al. (2011) distinguished between *exploitative entitlement* characterized by unrealistic expectations of preferential treatment and linked to higher psychopathy, neuroticism, and lower social commitment and *non-exploitative entitlement*, rooted in legitimate perceptions of fairness and associated with higher self-esteem.

**Table 1:** Selected entitlement measures across key domains.

Instrument	Authors (year)	Domain	Global vs. dimensions	# Items	Key dimensions	Best use case
PES – Psychological entitlement scale	Campbell et al. (2004)	Personality/general	Global (unidimensional), later refined	9	Grandiose vs. Vulnerable (Hart et al., 2020)	Most widely used; should be interpreted with moderators.
NPI – Narcissistic personality inventory (entitlement subscale)	Raskin and Terry (1988)	Narcissism/personality	Dimension within NPI	3	Entitlement facet	Captures entitlement as part of narcissism; pathological variant.
EAQ – Entitlement attitudes questionnaire	Żemojtel-Piotrowska et al. (2017)	Personality/social	Multidimensional	15	Active, Passive, Revenge	Multidimensional tool for general research; adaptive and maladaptive forms.
Lessard entitlement dimensions	Lessard et al. (2011)	Youth/personality	Two facets	18	Exploitative vs. Non-exploitative	Useful in youth samples; legitimate and exploitative entitlement.
SRE – Sense of relational entitlement	Tolmacz and Mikulincer (2011) and Tolmacz et al. (2021)	Romantic relationships	Multidimensional	33 (SRE); 15 (SRE-R)	Assertive, Inflated, Restricted	Valuable for studying entitlement in close relationships.
AES – Academic entitlement scale	Chowning and Campbell (2009)	Academic/students	Multidimensional	15	Externalized Responsibility, Entitled Expectations	Captures privilege consciousness in academic settings.
MEE – Measure of employee entitlement	Westerlaken et al. (2017)	Workplace/employees	Multidimensional	18	Reward-as-a-right, Self-focus, Excessive self-regard	Organizational contexts, capture mostly inflated attitudes.
EEQ – Emotional entitlement questionnaire	Laslo-Roth and George-Levi (2024)	Emotional domain	Multidimensional	15	Positive emotions, Negative emotions, Uncompromising entitlement	Useful in clinical contexts and research on mental health.

This differentiation laid the foundation for distinguishing normal from narcissistic entitlement (Ackerman and Donnellan, 2013), paralleling Pincus and Lukowitsky’s (2010) division between normal and pathological narcissism. Normal entitlement reflects legitimate claims based on self-worth and accomplishments, whereas narcissistic entitlement involves exaggerated and unrealistic demands, often at the expense of others.

Żemojtel-Piotrowska et al. (2017) introduced the Entitlement Attitudes Questionnaire (EAQ), which distinguishes three dimensions of entitlement. *Active entitlement* reflects rights-based claims supported by high self-esteem and an internal locus of control, and it has been linked to psychological well-being and self-enhancement values, while remaining unrelated to neuroticism, suggesting a non-pathological form (Piotrowski and Żemojtel-Piotrowska, 2009; Żemojtel-Piotrowska et al., 2013, 2015).

*Passive entitlement* refers to expectations that external institutions should meet one’s needs. It has been linked to lower self-esteem and an external locus of control, and was initially considered unrelated to narcissism (Piotrowski and Żemojtel-Piotrowska, 2009). Later work, however, suggested its dependency and communal focus align with vulnerable narcissism (Żemojtel-Piotrowska et al., 2015). Finally, *revenge entitlement* involves hostile claims for retribution and has been positively associated with narcissism and neuroticism and negatively with well-being, aligning with antagonistic narcissism through its exploitative and adversarial orientation (Żemojtel-Piotrowska et al., 2013, 2015; Ney and Fischweicher, 2021).

Żemojtel-Piotrowska et al. (2015) indicated that active entitlement was positively associated with subjective well-being and welfare. Confino et al. (2023), in a community-based sample of adults exposed to recent traumatic events, reported that active entitlement was positively linked to posttraumatic growth; individuals high on active entitlement appeared to hold a belief in their right to well-being, which may have facilitated proactive engagement in personal development.

Research has also shown that the PES, the classic measure of trait entitlement, is more complex than originally assumed. Hart et al. (2020) revised the PES to distinguish *grandiose entitlement*, linked to dominance, superiority, and antagonism, from *vulnerable entitlement*, associated with hypersensitivity, defensiveness, and perceived deprivation (Jauk et al., 2023). Similarly, Crowe et al. (2016), using cluster analyses of the PES, identified an *emotionally stable cluster of entitlement* (high self-esteem, emotional stability, positive affect) and an *emotionally vulnerable cluster of entitlement* (low self-esteem, high neuroticism, negative affect, childhood adversity and relational difficulties).

This distinction between grandiose and vulnerable forms parallels the well-established differentiation within narcissism, while underscoring entitlement as a related but distinct construct (Hart et al., 2020). Overall, Multidimensional models highlight that entitlement encompasses both adaptive forms - active, assertive, non-exploitative, and emotionally balanced - and maladaptive forms marked by rigid or exaggerated expectations and disregard for others.

### Manifestation of entitlement in different life domains

While entitlement has often been studied as a global trait, growing evidence shows that it also takes distinct forms across life domains, such as academic, workplace, relational and emotional contexts. This shift toward domain-specific perspectives has prompted the development of targeted measures and reveals that, much like findings in related constructs such as narcissism, the expression and consequences of entitlement vary across domains (Grosz et al., 2022; Orth et al., 2024). The following section outlines how entitlement manifests across different domains.

#### Relational

Relational entitlement (SRE) reflects beliefs about what one deserves in close relationships (Tolmacz and Mikulincer, 2011; Tolmacz et al., 2021). Three forms were identified by Tolmacz and Mikulincer (2011): *exaggerated entitlement*, reflecting unrealistic and disproportionate demands from others; *restricted entitlement*, marked by hesitation or fear of expressing one’s needs and representing the model’s major contribution by drawing attention to suppressed entitlement needs; and *assertive entitlement*, characterized by a balanced stance that integrates self-assertion with sensitivity to others.

Exaggerated and restricted forms of entitlement tend to undermine satisfaction and adjustment, with experimental findings supporting these distinctions (George-Levi et al., 2014; Candel and Turliuc, 2021; Tolmacz et al., 2021). Assertive entitlement shows mixed outcomes: it generally supports healthier functioning and self-esteem but may also contribute to relational strain (Candel and Turliuc, 2019). Later, healthy entitlement has been defined as low levels of both exaggerated and restricted forms. Evidence is mostly based on cross-sectional studies of non-clinical couples, though recent work has extended the measure beyond romantic relationships, supporting broader validation (Tolmacz et al., 2016, 2021, 2022).

#### Workplace

In organizational contexts, entitlement is often defined as the belief one deserves special rewards/advancement irrespective of performance, measured by the PES and adapted workplace scales (Jordan et al., 2017). Such expectations have been linked to negative outcomes, including poorer supervisor relations, performance deficits, and heightened workplace conflict (Fisk, 2010; Harvey and Dasborough, 2015). Recent evidence also highlights adaptive potential, as assertive or active entitlement has been linked to ambition, job involvement, and greater satisfaction, likely through mechanisms such as enhanced self-efficacy and fairness perceptions (Cohen et al., 2021; Dragova-Koleva, 2018).

#### Academic

Academic entitlement describes students’ expectations of favorable outcomes regardless of effort (Greenberger et al., 2008). Common in individualistic educational systems, it has been linked to reduced motivation, lower self-efficacy, and heightened frustration (Peirone and Maticka-Tyndale, 2017; Kinne et al., 2022). Some studies suggest that *general* entitlement can buffer negative effects of controlling parenting or predict more adaptive outcomes among students (Madison et al., 2025; Gao et al., 2025). Academic-specific entitlement, however, is largely studied as maladaptive and unidimensional, leaving its variability underexplored.

Laslo-Roth et al. (2024) showed that academic entitlement, defined as unrealistic expectations and demands within the academic setting, is associated with lower gratitude and greater loneliness, whereas active entitlement predicts the opposite pattern. Although entitlement may overlap with traits such as extraversion (Żemojtel-Piotrowska et al., 2017), the contrasting outcomes of these two forms point to the unique role of entitlement type, reflecting distinct orientations toward fairness and self-worth. Differentiating between forms of entitlement is therefore crucial for understanding their divergent implications for well-being.

#### Emotional

Emotional entitlement refers to beliefs about one’s right to experience particular emotional states (Laslo-Roth and George-Levi, 2024). It has been conceptualized in three forms: *entitlement to positive emotions*, the belief that one deserves to feel happy and fulfilled; *entitlement to negative emotions*, the belief that one has the right to freely experience sadness, anger, or fear; and *uncompromising emotional entitlement*, characterized by rigid expectations that others must meet one’s emotional needs, often leading to demands for validation, resentment, or vengefulness when expectations are unmet.

Entitlement to positive emotions was found to be associated with greater well-being, positive affect and less loneliness; uncompromising entitlement with distress; and entitlement to negative emotions shows mixed associations. Other studies confirm this structure (Huang et al., 2025), underscoring the dual potential of emotional entitlement for mental health.

### Sense of entitlement as a context-related construct

Research highlights the role of sociocultural context in activating entitlement beliefs. Żemojtel-Piotrowska et al. (2017) documented substantial cross-national variation in entitlement across 25 countries, underscoring the influence of cultural norms and values on entitlement beliefs. Similarly, Piff (2014) demonstrated that entitlement is shaped by structural factors such as social class.

Other studies have likewise highlighted the contextual responsiveness of entitlement. For example, studies have found that family stress can heighten entitlement (Cohen et al., 1996) and that mothers of children with developmental disabilities report higher entitlement which, in some cases, relates to greater well-being (George-Levi and Laslo-Roth, 2021). Experimental evidence similarly indicates that entitlement can be triggered by situational cues, such as exposure to stereotypes (Eschleman et al., 2017), receiving unearned rewards (Holderness et al., 2021), or recalling unfair treatment (Zitek et al., 2010).

Taken together, the evidence supports viewing entitlement as a context-activated construct shaped by the interplay between stable individual differences and situational factors. Yet the scarcity of experimental research and limited attention to cultural differences leave the mechanisms of activation insufficiently understood.

### Moderating factors shaping adaptive and maladaptive expressions of entitlement

Once activated, entitlement does not unfold uniformly. Its expression and consequences depend on moderating conditions that regulate how individuals interpret and respond to unmet expectations (Grubbs and Exline, 2016). For example, Stronge and Sibley (2021) found that psychological entitlement, as measured by the PES, was generally associated with poorer well-being. However, this association did not hold for young adults, suggesting that in this age group, entitlement may serve a more adaptive or developmentally normative role.

Moderating factors are therefore central mechanisms that determine whether entitlement is derailed into cycles of antagonism and conflict or redirected into proactive, adaptive forms of self-assertion, with growing evidence that they buffer negative effects and may even reverse them. At the interpersonal level, responsiveness in close relationships, supportive organizational climates, and perceptions of mutual obligation provide external validation that mitigates dissonance and prevents entitlement from escalating into negative outcomes (Bar-Kalifa et al., 2016; Schwarz et al., 2023; Chen et al., 2023).

At the intrapersonal level, strengths such as self-compassion (Yang et al., 2025), authenticity (Sun et al., 2022), and hope (George-Levi and Laslo-Roth, 2021) act as internal regulators that buffer the negative effects of unmet needs among highly entitled individuals. For instance, high entitlement predicted greater well-being when accompanied by hope, defined as goal-directed thinking that integrates agency and pathways, but the opposite trend emerged under low hope (George-Levi and Laslo-Roth, 2021). These mechanisms may work by reframing entitlement claims as expressions of legitimate self-worth, thereby reducing defensiveness and channeling energy into adaptive goal pursuit (Yang et al., 2025). Similarly, low narcissism and effective self-monitoring promote adaptability and reduce exploitative tendencies in entitled individuals (Klimchak et al., 2016; Langerud and Jordan, 2020).

Most importantly, it is the interaction between intrapersonal and interpersonal resources that may ultimately shape entitlement’s trajectory (Kristof-Brown and Guay, 2011). Internal strengths may prepare individuals to adjust expectations, but without supportive environments entitlement can remain volatile. Conversely, external responsiveness may legitimize entitlement claims, but in the absence of intrapersonal regulation it risks reinforcing rigid or exploitative patterns.

## Discussion

In contemporary societies, where rights and personal needs are increasingly emphasized, entitlement represents both an opportunity and a risk. The present mini-review, while selective rather than systematic, highlights ongoing debates over whether entitlement is a distinct construct or simply overlaps with traits such as narcissism. Emerging evidence indicates that while entitlement is strongly related to narcissism, it reflects unique beliefs about deservingness that extend beyond narcissism and other personality traits (Ackerman and Donnellan, 2013).

### Advances in definitions of entitlement

The findings demonstrate that the definition of entitlement has expanded beyond its traditional portrayal as a purely negative trait. Whereas earlier literature emphasized excessive and exploitative entitlement, demanding more from others regardless of one’s own input, recent models highlight its multi-faceted, context-sensitive nature. Entitlement may manifest in active-assertive or equality-based forms, reflecting a legitimate sense of deservingness that can generate positive outcomes (Żemojtel-Piotrowska et al., 2017), as well as in restricted forms, where the suppression of entitlement needs proves detrimental (Tolmacz et al., 2021).

**Figure 1:**
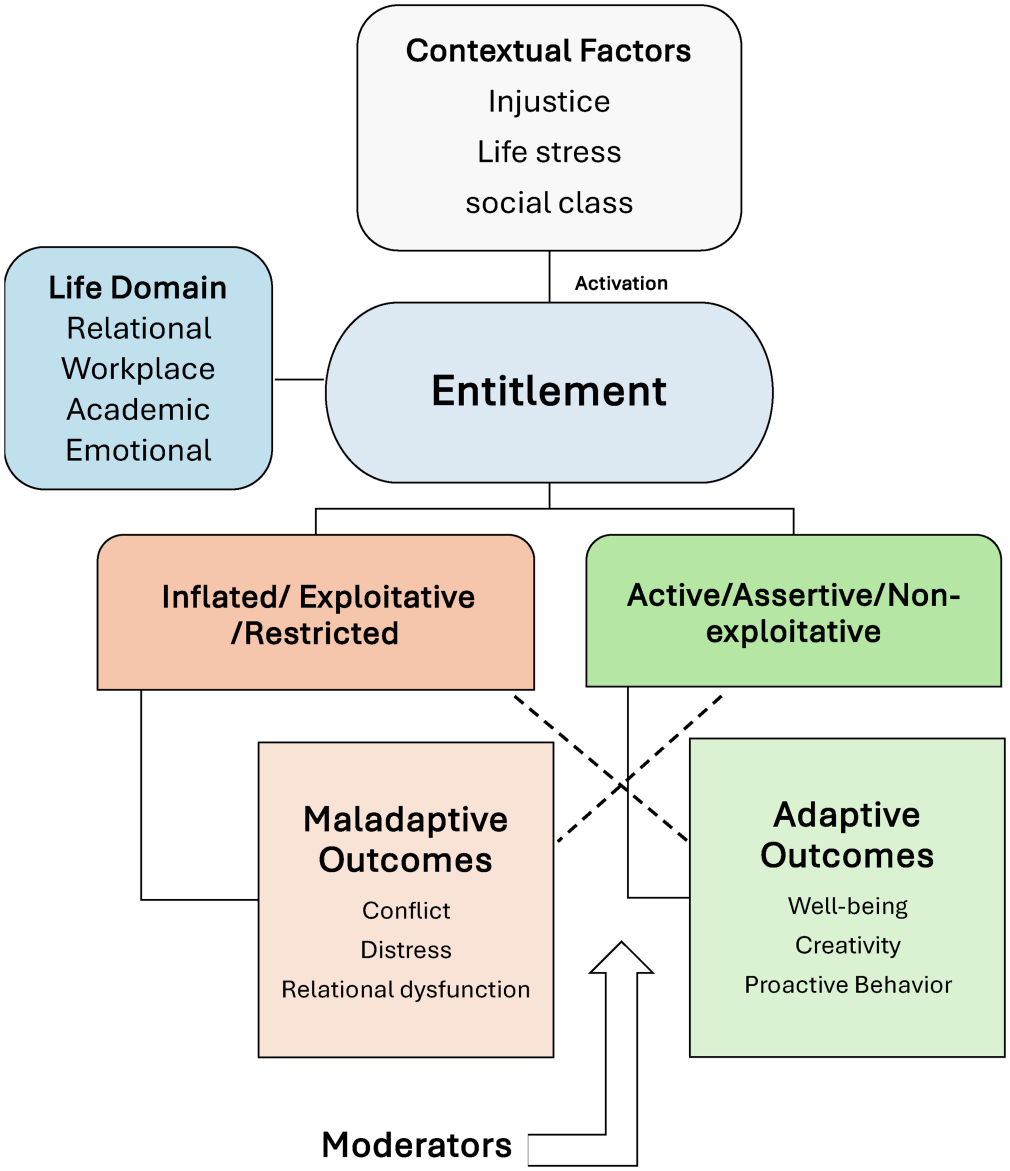
A simplified framework of entitlement. Contextual factors activate entitlement. Entitlement is then expressed across different life domains and differentiates into adaptive forms (active/assertive/non-exploitative) or maladaptive forms (inflated/exploitative/restricted). These forms can generate either positive outcomes such as well-being and creativity or negative outcomes such as conflict and distress, with the direction shaped by intrapersonal and interpersonal moderators.

This broader view suggests that the impact of entitlement depends on how it is defined, whether as a unidimensional excess or a multidimensional construct, and how it is shaped by personal resources, situational demands, and cultural contexts. Contextual factors may activate entitlement beliefs, while moderators determine whether these beliefs are expressed adaptively or maladaptively. Across relational, academic, workplace, and emotional domains, entitlement can thus foster motivation and resilience or fuel conflict and dysfunction. A synthesized, integrative framework (Figure 1) offers a roadmap for clarifying these dynamics and advancing future research.

### Toward integrative models

Personality and social perspectives on entitlement, once viewed as competing, are now better understood as complementary, underscoring the need for integrative models that account for both stable trait-like dimensions and context-dependent expressions of entitlement. Such integration is evident in Tomlinson’s (2013) model, which frames entitlement as both a dispositional trait and a context-dependent belief shaped by environmental factors, and in the schema model, which conceptualizes entitlement as a self-regulatory process rooted in early experiences and activated when perceptions of fairness, self-worth, or control are threatened (Bach et al., 2018).

### Limitations and open questions

A central challenge in developing integrative models is to identify the processes that determine when entitlement functions adaptively versus maladaptively. Active forms may foster agency, fairness perceptions, and constructive self-assertion, whereas inflated or revengeful forms often fuel resentment and conflict (Żemojtel-Piotrowska et al., 2016). Although moderators such as hope and supportive climates show promise, evidence on mediating mechanisms remains scarce. Progress is further hampered by inconsistent measures and reliance on cross-sectional designs, which obscure developmental and causal pathways (Peng et al., 2024; Zitek and Vincent, 2015). Open questions include whether entitlement truly enhances well-being via agency and fairness perceptions, or whether individuals with greater well-being are simply more inclined toward active entitlement, and how contextual, cultural and developmental factors shape entitlement expression over time.

### Best practice recommendations and future directions

The PES remains the most validated and widely used measure, particularly effective for assessing excessive or inflated entitlement. However, unidimensional tools such as the PES, which capture only inflated beliefs, require cautious interpretation and are best considered alongside intrapersonal and interpersonal moderators. Recent literature supports a multidimensional approach. The EAQ offers a promising framework by distinguishing active, passive, and revenge dimensions of general entitlement, while domain-specific scales are preferable when a particular context is central. Table 1 provides an integrated overview of existing instruments and their recommended applications.

Best practice recommendations vary across domains. In academia, there is a need to move beyond the narrow focus on excessive academic entitlement by adopting multidimensional tools; interventions should promote effort-based attributions, egalitarian values, and an internal locus of control (Peng et al., 2024). In the workplace, progress has already been made by applying multi-faceted models and moderators to link entitlement with motivation and organizational outcomes. Practices such as realistic job previews, transparent communication, performance-based rewards, and supportive climates have been shown to channel entitlement into commitment and proactivity (Yang et al., 2024).

In relational contexts, research highlights the importance of attending not only to inflated entitlement but also to restricted entitlement, which undermines relationship quality. Recalibrating unrealistic expectations through communication, emotional expression, and equity restoration can strengthen relational functioning (Tolmacz et al., 2021). In the emotional domain, which is still emerging, the EEQ provides a promising tool for monitoring mental health and therapeutic change: declines in uncompromising entitlement indicate greater flexibility, while increases in entitlement to diverse emotions may reflect enhanced emotional acceptance (Huang et al., 2025).

## Conclusion

Entitlement shows its dark sides when inflated and dismissive of others, and its bright sides when grounded in self-worth, agency, and fairness (Lin et al., 2023). While entitlement may function as a privilege in some contexts, it can also serve as a vital resource - for disadvantaged groups seeking fairness, for individuals safeguarding their own or loved ones’ needs, and in affirming the right to experience emotions or anticipate positive outcomes. Best practice is to distinguish forms and contexts, use multidimensional measures where possible, interpret unidimensional tools with attention to moderators, and tailor assessments to specific domains. This integrative approach acknowledges both the adaptive potential of entitlement and its risks.
